# Treatment With Calcineurin Inhibitor FK506 Attenuates Noise-Induced Hearing Loss

**DOI:** 10.3389/fcell.2021.648461

**Published:** 2021-03-12

**Authors:** Zu-Hong He, Song Pan, Hong-Wei Zheng, Qiao-Jun Fang, Kayla Hill, Su-Hua Sha

**Affiliations:** Department of Pathology and Laboratory Medicine, Medical University of South Carolina, Charleston, SC, United States

**Keywords:** noise-induced hearing loss, calcineurin inhibitor, nuclear factor of activated T-cells isoform c4, reactive oxygen species, autophagy

## Abstract

Attenuation of noise-induced hair cell loss and noise-induced hearing loss (NIHL) by treatment with FK506 (tacrolimus), a calcineurin (CaN/PP2B) inhibitor used clinically as an immunosuppressant, has been previously reported, but the downstream mechanisms of FK506-attenuated NIHL remain unknown. Here we showed that CaN immunolabeling in outer hair cells (OHCs) and nuclear factor of activated T-cells isoform c4 (NFATc4/NFAT3) in OHC nuclei are significantly increased after moderate noise exposure in adult CBA/J mice. Consequently, treatment with FK506 significantly reduces moderate-noise-induced loss of OHCs and NIHL. Furthermore, induction of reactive oxygen species (ROS) by moderate noise was significantly diminished by treatment with FK506. In agreement with our previous finding that autophagy marker microtubule-associated protein light chain 3B (LC3B) does not change in OHCs under conditions of moderate-noise-induced permanent threshold shifts, treatment with FK506 increases LC3B immunolabeling in OHCs after exposure to moderate noise. Additionally, prevention of NIHL by treatment with FK506 was partially abolished by pretreatment with LC3B small interfering RNA. Taken together, these results indicate that attenuation of moderate-noise-induced OHC loss and hearing loss by FK506 treatment occurs not only *via* inhibition of CaN activity but also through inhibition of ROS and activation of autophagy.

## Introduction

Noise-induced hearing loss (NIHL) acquired from military duty, industrial occupations, and recreation and leisure activities is the most common occupational disease in the US and probably worldwide (Neitzel and Fligor, 2019; Themann and Masterson, 2019). Loss of sensory hair cells in the cochlea, with outer hair cells (OHCs) being more vulnerable than inner hair cells (IHCs), has been well documented in humans and various animal models as a cause of permanent threshold shifts (PTS or permanent hearing loss) (Sha and Schacht, 2017; Wang and Puel, 2018). Although the molecular events occurring after noise exposure are highly complex, the notion of overload of calcium in the endolymph and hair cells, accumulation of reactive oxygen species (ROS), and increased cytokines contributing to the pathogenesis of noise-induced loss of sensory hair cells is well accepted (Ikeda and Morizono, 1988; Fridberger et al., 1998; Ohlemiller et al., 1999; Yamashita et al., 2004; Fujioka et al., 2006; Chen et al., 2012; Hill et al., 2016; Dhukhwa et al., 2019; Fettiplace and Nam, 2019).

Calcineurin (CaN/PP2B) belongs to the protein phosphatase 2B family of Ca^2+^/calmodulin-dependent protein phosphatases and is activated by binding between Ca^2+^ and calmodulin (Hashimoto et al., 1990; Morioka et al., 1999). The activation of CaN may contribute to hair cell death as treatment with CaN inhibitor FK506 (tacrolimus) attenuates noise and aminoglycoside-induced hair cell loss and hearing loss (Minami et al., 2004; Uemaetomari et al., 2005; Bas et al., 2012), but the underlying mechanisms are not fully understood. FK506 forms a complex with binding protein FKBP12 (FK506-binding protein), which binds to a common composite surface made up of residues from the catalytic subunit of CaN (Ke and Huai, 2003) and, in turn, inhibits CaN activity. Nuclear factor of activated T-cells (NFAT), a downstream target of CaN, is an attractive candidate as the executor of CaN's detrimental effects. Five NFAT family members, NFAT1 (NFATp or NFATc2), NFAT2 (NFATc1), NFAT3 (NFATc4), NFAT4 (NFATc3 or NFATx), and NFAT5, have been identified (Rao et al., 1997; Crabtree and Olson, 2002). Following increases in intracellular Ca^2+^, NFATs are dephosphorylated by CaN and subsequently form a complex with CaN that is translocated from the cytoplasm to the nucleus (Shibasaki et al., 1996). Several *in-vivo* experiments show that the use of constitutively active CaN leads to the translocation of NFAT3 into the nuclei in the brain following ischemia (Shioda et al., 2006, 2007), in Alzheimer's disease (Abdul et al., 2009), and in neuronal apoptosis (Shioda et al., 2007). Therefore, NFAT3 can be used as a marker of CaN activity.

Recently, a report showed that *Nfatc3* (*NFAT3*) deficiency in mice attenuates ototoxicity by suppressing TNF-mediated hair cell apoptosis (Zhang et al., 2019). In fact, overproduction of both ROS and cytokines has been well documented in noise trauma with loss of OHCs (Yamane et al., 1995; Shi et al., 2003; Yamashita et al., 2005; Fujioka et al., 2006; Le Prell et al., 2007; Dhukhwa et al., 2019; Fetoni et al., 2019; Frye et al., 2019). It is speculated that ROS and inflammation have a complex interplay (Fetoni et al., 2019). Additionally, autophagy dysfunction has been suggested to induce several pathological states, like cancer, inflammation, neurodegenerative diseases, and metabolic disorders (Levine and Kroemer, 2008; Arroyo et al., 2013; Ryter et al., 2014). In the inner ear, the lower levels of oxidative stress induced by temporary threshold shift (TTS) noise exposure or lower doses of aminoglycoside treatment inhibit apoptosis and promote hair cell survival *via* autophagy (Yuan et al., 2015; He et al., 2017). On the other hand, excessive activation of autophagy may induce cell death (Kroemer and Levine, 2008; Bandyopadhyay et al., 2014; Wu et al., 2020b). Interestingly, FK506 also activates the autophagy system by binding to the V-ATPase catalytic subunit A in neuronal cells (Kim et al., 2017; Wang et al., 2017). Autophagy, as a major cellular self-protection mechanism, plays a role in adapting cells and organs to changing micro-environments by eliminating intracellular components and potentially harmful molecules and organelles. Therefore, we speculate that treatment with FK506 not only inhibits CaN but also inhibits ROS and activates autophagy for prevention of NIHL.

In this study, we investigated the attenuation of noise-induced OHC loss and hearing loss by FK506 *via* inhibition of CaN activity and ROS accumulation and the promotion of autophagy using immunohistochemistry and small interfering RNA silencing (siRNA) techniques in adult CBA/J mice. In agreement with previous results (Minami et al., 2004; Uemaetomari et al., 2005; Bas et al., 2012), our data support the notion that treatment with FK506 prevents NIHL.

## Materials and Methods

### Animals

Male CBA/J mice at 10 weeks of age were purchased from The Jackson Laboratory. All mice had free access to water and a regular mouse diet (irradiated lab diet #5V75) and were kept at 22 ± 1°C under a standard 12:12-h light–dark cycle to acclimate for at least 1 week before conducting baseline auditory brainstem response (ABR) measurements. CBA/J mice at the age of 12 weeks were exposed to noise. The mice were euthanized 2 weeks after auditory functional measurement for hair cell morphological analysis or 1–3 h after noise exposure for immunolabeling of protein expression in OHCs. All mice were specific pathogen-free and housed in the animal facility with controlled noise levels [below 60 dB sound pressure level (SPL)] in the Children's Research Institute at the Medical University of South Carolina. All research protocols were approved by the Institutional Animal Care and Use Committee at MUSC (protocol # IACUC-2019-00752). Animal care was under the supervision of the Division of Laboratory Animal Resources at MUSC. A randomized two- to three-animal block allocation was employed to assign animals to different experimental groups with three to four repetitions for each experiment. No animals were excluded or died during the experiments.

### Noise Exposure

Unrestrained male CBA/J male mice at the age of 12 weeks (one mouse per stainless steel wire cage, ~9 cm^3^) were exposed to broadband noise (BBN) with a frequency spectrum of 2–20 kHz at 101–103 dB SPL for 2 h to induce permanent threshold shifts (PTS) at 16 and 32 kHz with loss of OHCs by 14 days after the noise exposure, referred to as our moderate-PTS-noise conditions. The mice were exposed to BBN at 106–108 dB SPL for 2 h to induce severe permanent threshold shifts (sPTS) at 8, 16, and 32 kHz with loss of sensory hair cells including both OHCs and IHCs by 14 days after the noise exposure, referred to as our sPTS-noise conditions. Noise exposures were conducted in the morning (between 9 and 11 a.m.) to avoid confounding influences of circadian rhythm on hearing function. The sound exposure chamber was fitted with a loudspeaker (model 2450H + 2385A; JBL) driven by a power amplifier (model XLS 202D; Crown Audio) fed from a CD player (model CD-200; Tascam TEAC American). Audio CD sound files were created and equalized with audio editing software (Audition 3; Adobe Systems, Inc.). The background sound intensity of the environment surrounding the cages was 65 dB as measured with a sound level meter (model 1200; Quest Technologies). Noise sound pressure level calibration was performed immediately before each exposure session. The sound levels were calibrated with a Bruel and Kjaer condenser microphone, allowing precise calibration and monitoring of the sound exposure. The noise level varied by a maximum of 1–2 dB across the measured sites within the exposure chamber. The sound levels for noise exposure were measured with a sound level meter at multiple locations within the sound chamber to ensure uniformity of the sound field and measured before and after exposure to ensure stability. Control mice were kept in silence (without use of the loudspeaker) within the same chamber for 2 h.

### Drug Administration *via* Intra-Peritoneal Route

FK506 (tacrolimus, #F4679) was purchased from Sigma-Aldrich, dissolved in dimethyl sulfoxide (DMSO) as a stock solution (20 mg/ml), and stored at −20°C. The stock solution was diluted with 0.9% saline solution immediately before injections. Initially, we tested two doses of FK506 (3 and 5 mg/kg) for prevention of NIHL based on prior literature (Uemaetomari et al., 2005). Since 5 mg/kg of FK506 attenuated NIHL, we used 5 mg/kg for the rest of the experiments. For immunohistochemistry, each animal received a total of three intraperitoneal (IP) injections of FK506 at a dose of 5 mg/kg per injection. The vehicle control mice received the same volume of DMSO (0.1%) in saline. Three IP injections were administered 24 h before, 1 h before, and immediately after the noise exposure. The mice used for the experiments to observe the effects of treatment on ABR thresholds received two additional IP injections on the day following the noise exposure (a.m. and p.m.).

### Auditory Brainstem Response Measurements

ABRs were measured before and 2 weeks after the noise exposure. The mice were anesthetized with an IP injection of a mixture of ketamine (100 mg/kg) and xylazine (10 mg/kg). After anesthesia, the mice were placed in a sound-isolated and electrically shielded booth (Acoustic Systems). Body temperature was monitored and maintained near 37°C with a heating pad. Acoustic stimuli were delivered monaurally to a Beyer earphone attached to a customized plastic speculum inserted into the ear canal. Subdermal electrodes were inserted at the vertex of the skull (active), mastoid region under the left ear, and mastoid region under the right ear (ground). ABRs were measured at 8, 16, and 32 kHz. Tucker-Davis Technologies (TDT) System III hardware and SigGen/Biosig software were used to present the stimuli (15-ms-duration tone bursts with 1-ms rise–fall time) and record the response. Up to 1,024 responses were averaged for each stimulus level. ABR wave II was used to determine the ABR thresholds for each frequency. Thresholds were determined for each frequency by reducing the intensity in 10-dB increments and then in 5-dB steps near the threshold until no organized responses were detected. Thresholds were estimated between the lowest stimulus level where a response was observed and the highest level without response. All ABR measurements were conducted by the same experimenter. The ABR values were assigned by an expert who was blinded to the treatment conditions.

### Intra-Tympanic Delivery of LC3B siRNA *in vivo*

LC3B siRNA (siLC3B, Thermofisher, 4390771) or scrambled siRNA (siControl, Thermofisher, 4390844) was delivered locally *via* intra-tympanic application as previously described (Chen et al., 2013; Oishi et al., 2013). Briefly, after anesthesia, a retroauricular incision (left ear) was made to approach the temporal bone. The otic bulla was identified ventral to the facial nerve, and a shallow hole was made in the thin part of the otic bulla with a 30-G needle and enlarged with a dental drill to a diameter of 2 mm in order to visualize the round window. A customized sterile micro-medical tube was inserted into the hole just above the round window niche (RWN) to slowly deliver 10 μl (0.6 μg) of pre-designed siLC3B or siControl to completely fill the mouse RWN. After the siRNA was delivered, the hole was covered with the surrounding muscle. Finally, the skin incision was closed with tissue adhesive. The animal was allowed to rest in surgical position for an additional 30–60 min before waking from anesthesia. About 72 h after siRNA delivery, the animals were exposed to noise for 2 h. Based on our previous experiments, local intra-tympanic delivery of siRNA results in a temporary elevation of thresholds that completely recovers to baseline after 48 h (Oishi et al., 2013; Zheng et al., 2014; Yuan et al., 2015). Therefore, noise exposure was performed near 72 h after siRNA delivery.

### Immunocytochemistry for Cochlear Surface Preparations

We have followed a procedure as previously described in detail (Fang et al., 2019). Briefly, the temporal bones were removed and perfused locally with a solution of 4% paraformaldehyde in phosphate-buffered saline (PBS), pH 7.4, and kept in this fixative overnight at 4°C. Between each step, the cochlear samples were washed at least three times with PBS for 5–10 min each wash. After decalcification with 4% sodium ethylenediaminetetraacetic acid solution (adjusted with HCl to pH 7.4) for 3 days at 4°C, the cochleae were micro-dissected into three turns (apex, middle, and base) and adhered to 10-mm round coverslips (Microscopy Products for Science and Industry, #260367) with Cell-Tak (BD Biosciences, #354240). The specimens were first permeabilized in 2% Triton X-100 solution and then blocked with 10% normal goat serum for 30 min each step at room temperature, followed by incubation with primary antibodies: monoclonal mouse anti-calcineurin (BD Biosciences #610260), monoclonal rabbit anti-NFAT3 at 1:50 (Sigma-Aldrich #SAB4501982), rabbit polyclonal anti-4 hydroxynonenal (4-HNE) at 1:100 (Abcam, #46545), and rabbit anti-LC3B at 1:200 (Cell Signaling Technology, #2775) at 4°C for 48 h. The specimens were then incubated with the Alexa-Fluor-488-conjugated or Alexa-Fluor-594-conjugated secondary antibody at a concentration of 1:200 at 4°C overnight and followed by incubation with propidium iodide (PI) or phalloidin for 1 h at room temperature in darkness. Control incubations were routinely processed without primary antibody treatments.

Surface preparations for counting of hair cells were incubated with Myosin-VIIa (Proteus Biosciences, #25-6790, 1:200) at 4°C overnight and then incubated overnight at 4°C with secondary antibody (biotinylated goat anti-rabbit) at a 1:100 dilution. The specimens were then incubated in ABC solution (Vector Laboratories, PK-4001) overnight followed by incubation in 3,3′-diaminobenzidine (DAB) for 3 h as necessary for sufficient staining intensity. Finally, the specimens were washed to stop the DAB reaction.

After at least three final washes with PBS, all immunolabeling samples (already on round coverslips) were mounted by adding 8 μl mounting agent (Fluoro-gel with Tris buffer, Electron Microscopy Sciences, #17985-10), sandwiched with another round coverslip, and placed on a microscope slide. Finally, the edges were sealed with nail polish. The immunolabeled images were taken with a ×63-magnification lens under identical Z-stack conditions using Zeiss LSM 880.

### Semi-quantification of the Immunolabeling Signals From Outer Hair Cells or Outer Hair Cell Nuclei of Surface Preparations

Immunohistochemistry is well-accepted as a semi-quantitative methodology when used with careful consideration of the utility and semi-quantitative nature of these assays (Taylor and Levenson, 2006; Walker, 2006). Immunolabeling for CaN, NFAT3, 4-HNE, and LC3B was semi-quantified from original confocal images with eight-bit grayscale values, each taken with a ×63-magnification lens under identical conditions and equal parameter settings for laser gains and photomultiplier tube gains within linear ranges of the fluorescence using Image J software (National Institutes of Health, Bethesda, MD). The cochleae from the different groups were fixed and immunolabeled simultaneously with identical solutions and processed in parallel. All surface preparations were counterstained with phalloidin (green) or propidium iodide (red) to identify the comparable parts of the OHC or OHC nuclei in confocal images. The regions of interest of individual OHCs or OHC nuclei were outlined with the circle tool based on phalloidin or PI staining. The immunolabeling in grayscale in OHCs was measured in the upper basal region of surface preparations (corresponding to sensitivity to 22–32 kHz) in 0.12-mm segments, each containing about 60 OHCs. The intensity of the background was subtracted, and the average grayscale intensity per cell was then calculated. For each repetition, the relative grayscale value was determined by normalizing the ratio to control. Since there were no significant changes in all assessed immunolabeling in the apex and middle regions of cochlear OHC or OHC nuclei when assessed 1–3 h after the completion of the noise exposure, we performed only semi-quantification of the immunolabeling signals from OHC or OHC nuclei in the basal turn (corresponding to sensitivity to 22–32 kHz). This procedure provided semi-quantitative measurements that are not confounded by protein expression in other cell types of the cochlea.

### Hair Cell Counts

Images from the apex through the base of the Myosin-VIIa-labeled and DAB-stained surface preparations were captured using a ×20 lens on a Zeiss microscope. The lengths of the cochlear epithelia were measured and recorded in millimeters. Mapping of frequencies as a function of distance along the entire length of the cochlear spiral was calculated with the equation [*d* (%) = 156.5 – 82.5 × log (*f*)] from Müller's paper (Muller et al., 2005). The results are in agreement with the literature (Viberg and Canlon, 2004). OHCs were counted from the apex to the base along the entire length of the mouse cochlear epithelium. The percentage of hair cell loss in each 0.5-mm length of epithelium was plotted as a function of the cochlear length as a cytocochleogram (Zheng et al., 2014).

### Cell Culture, LC3B Silencing, and Protein Extraction

HEI-OC1, an inner ear cell line, was kindly provided by Dr. Federico Kalinec at UCLA Health. HEI-OC1 cells were seeded in six-well dishes to about 2 × 10^5^ cells/well and cultivated in Dulbecco's modified Eagle's medium (DMEM, Invitrogen, #11965-084) containing 4.5 g/l glucose and 10% fetal bovine serum (FBS) (Fisher Scientific, #16000044) in a humidified incubator (33°C, 10% CO_2_, 95% humidity). For LC3B siRNA transfection into cells, the same LC3B siRNA (Thermofisher, 4390771) or scrambled siRNA Control (Thermofisher, 4390844) was used as in *in vivo* experiments. Lipofectamine^TM^ RNAIMAX Reagent (Invitrogen, 13778075) was used for the transfection. The cells were seeded in six-well dishes and cultivated in DMEM containing FBS until reaching 70% confluence; the medium was then replaced with serum-free DMEM before transfection. The cells were transfected with siLC3B or siControl according to the manufacturer's instructions. The transfections lasted for 6 h, and the medium was replaced with fresh DMEM containing FBS and cultured for another 42 h. The cells were digested with 0.25% trypsin. The collected cells were transferred to a 15-ml conical tube (Corning, #430052) and centrifuged at 500 × *g* for 5 min, the medium was decanted, and the cells were washed with 1 ml of PBS (Invitrogen, #20012). After removing the PBS, total protein was extracted using RIPA buffer (Sigma-Aldrich, #R0278) contained phosphatase inhibitor (Sigma-Aldrich, #04906845001) following the provided instructions. Finally, total protein was stored at −80°C after quantification. In this study, the HEI-OC1 cells used were between 10 and 20 culture passages.

### Western Blot Analysis

Protein samples (30 μg) were separated by sodium dodecyl sulfate–polyacrylamide gel electrophoresis. After electrophoresis, the proteins were transferred onto a nitrocellulose membrane (Pierce) and blocked with 5% solution of nonfat dry milk in PBS−0.1% Tween 20 (PBS-T). The membranes were incubated with monoclonal rabbit anti-LC3B (Cell Signaling Technology, #3868, 1:200) at 4°C overnight and then washed three times (10 min each) with PBS-T buffer. The membranes were then incubated with the appropriate secondary antibody at a concentration of 1:2,500 for 1 h at room temperature. Following extensive washing of the membrane, the immunoblot bands were visualized by SuperSignal West Dura Extended Duration Substrate or Pierce® ECL Western Blotting Substrate (Thermo Scientific). The membranes were then stripped and relabeled for GAPDH (Cell Signaling Tech., #5174, 1:3,000) as a sample loading control.

Western blot bands were scanned by the LI-COR Odyssey Fc imaging system and analyzed using Image J software. First, the background staining density for each band was subtracted from the band density. Next, the probing protein/GAPDH ratio was calculated from the band densities run on the same gel to normalize for differences in protein loading. Finally, the difference in the ratio of the control and experimental bands was tested for statistical significance.

### Statistical Analyses

Data were analyzed using SYSTAT 8.0 and GraphPad 5.0 software for Windows. Biological sample sizes were determined based on the variability of measurements and the magnitude of the differences between groups as well as experience from our previous studies, with stringent assessments of difference. Data of OHC loss along the length of the cochlear spiral were analyzed with one-way repeated-measures analysis of variance (ANOVA) with *post hoc* tests using SYSTAT 8.0. The rest of the analyses were done using GraphPad 5.0. Differences with multiple comparisons were evaluated by one-way ANOVA with multiple comparisons. Differences for single-pair comparisons were analyzed using two-tailed unpaired Student's *t-*tests. Data for relative ratios of single-pair comparisons were analyzed with one-sample *t-*tests. A *p*-value < 0.05 was considered statistically significant. Data are presented as means ± SD or SEM based on the sample size and variability within groups. Sample sizes are indicated for each figure.

## Results

### Noise Increases Immunolabeling for CaN in Outer Hair Cells and NFAT3 in Outer Hair Cell Nuclei

**Figure 1:**
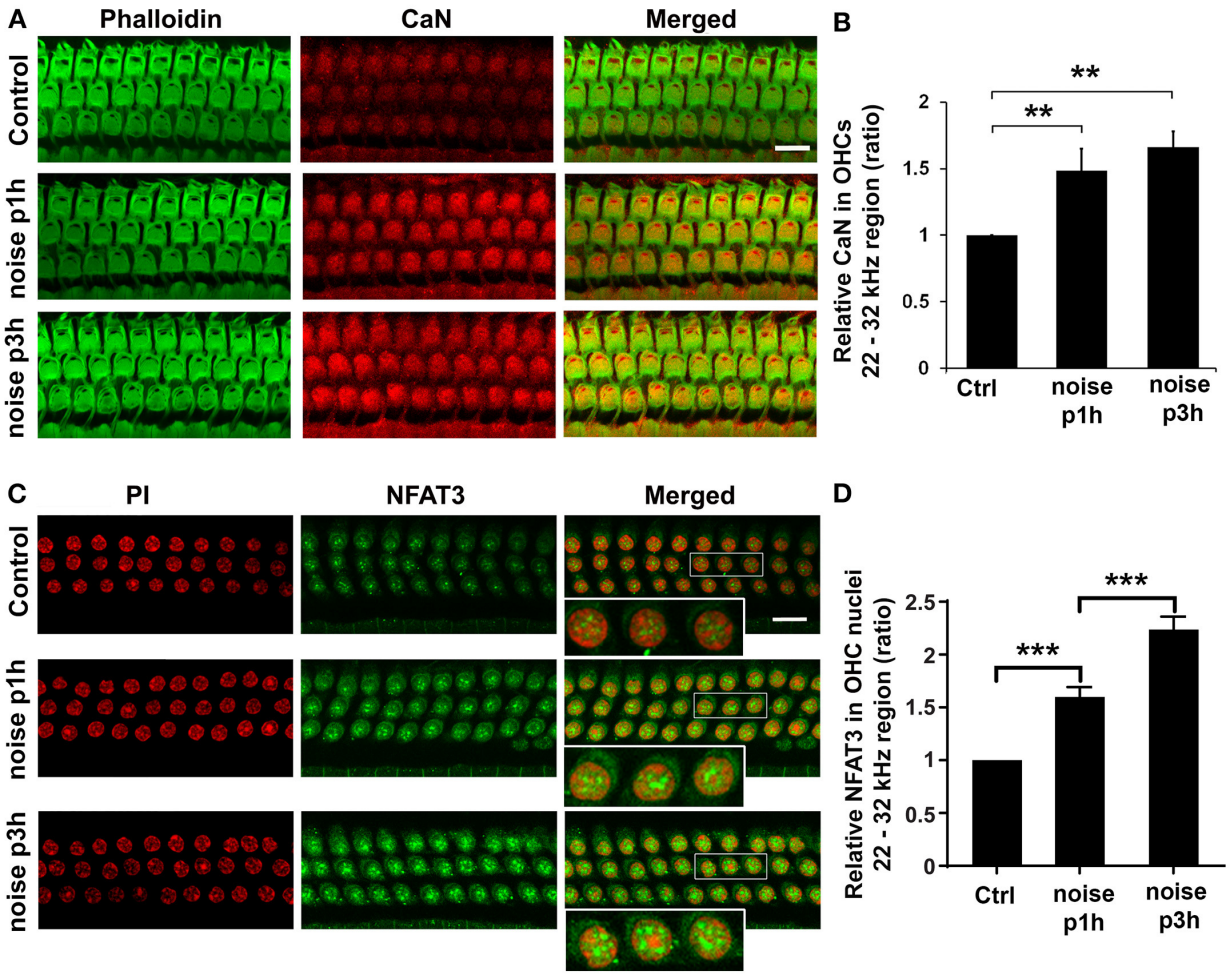
Noise exposure increases CaN in outer hair cells (OHCs) and NFAT3 in OHC nuclei. **(A)** Surface preparations of the cochlear epithelium show that immunolabeling for CaN (red) in OHCs was stronger in noise-exposed mice examined 1 and 3 h after the completion of exposure compared to control mice without exposure. Green, phalloidin-labeled sensory hair cells. Images were taken from the basal turn corresponding to sensitivity to 22–32 kHz, and each figure is representative of five individual mice for each condition. Scale bar = 10 μm. **(B)** Quantification of relative CaN immunolabeling intensity in grayscale in OHCs normalized to control mice confirms significant increases. Data are presented as means ± SD; *n* = 5 for each condition with one ear analyzed per mouse; **p < 0.01. **(C)** Surface preparations show that NFAT3 immunolabeling was stronger in OHC nuclei 1 h after, followed by an even greater increase 3 h after exposure. For better visualization, three OHC nuclei were enlarged of the merged panels. This figure is representative of one ear per mouse in five individual mice in each group. Scale bar = 10 μm. **(D)** Quantification of NFAT3 immunolabeling intensity in grayscale in OHC nuclei confirms a significant increase 1 h after and a further increase 3 h after exposure. Data are presented as means ± SD; *n* = 5 for each condition, ***p < 0.001.

To determine whether CaN and NFAT3 are linked to noise-induced outer hair cell death, we first assessed the expression of CaN and NFAT3 in sensory hair cells in response to PTS noise because NFAT, a downstream target of CaN, is an attractive candidate for the pathogenesis of the underlying detrimental effects of CaN (Shioda et al., 2006, 2007). Immunolabeling for CaN (red) on surface preparations increased in OHCs 1 and 3 h after noise exposure compared to control mice without exposure (Figure 1A). A semi-quantitative analysis of CaN immunolabeling intensity converted to grayscale in OHCs showed a statistically significant increase, with a ratio of control to 1 and 3 h post-exposure of 1:1.48 (*t*_4_ = 6.6388, *p* = 0.0027) and 1:1.60, respectively (*t*_4_ = 12.8305, *p* = 0.0002, Figure 1B). Although the immunolabeling for CaN remained stably elevated 3 h after exposure, there was no difference between 1 and 3 h post-exposure. Immunolabeling for NFAT3 in OHC nuclei appeared stronger and more punctate 1 and 3 h after exposure (Figure 1C). Semi-quantification of immunolabeling for NFAT3 (converted to grayscale) in OHC nuclei increased when examined 1 h after (*t*_3_ = 6.3, *p* = 0.0078) and continued to increase 3 h after (*t*_6_ = 4.1, *p* = 0.0065) the exposure (Figure 1D). These results support the notion that NFAT3 acts as an indicator of CaN activity.

### Treatment With FK506 Attenuates Noise-Induced Hearing Loss and Outer Hair Cell Loss

**Figure 2:**
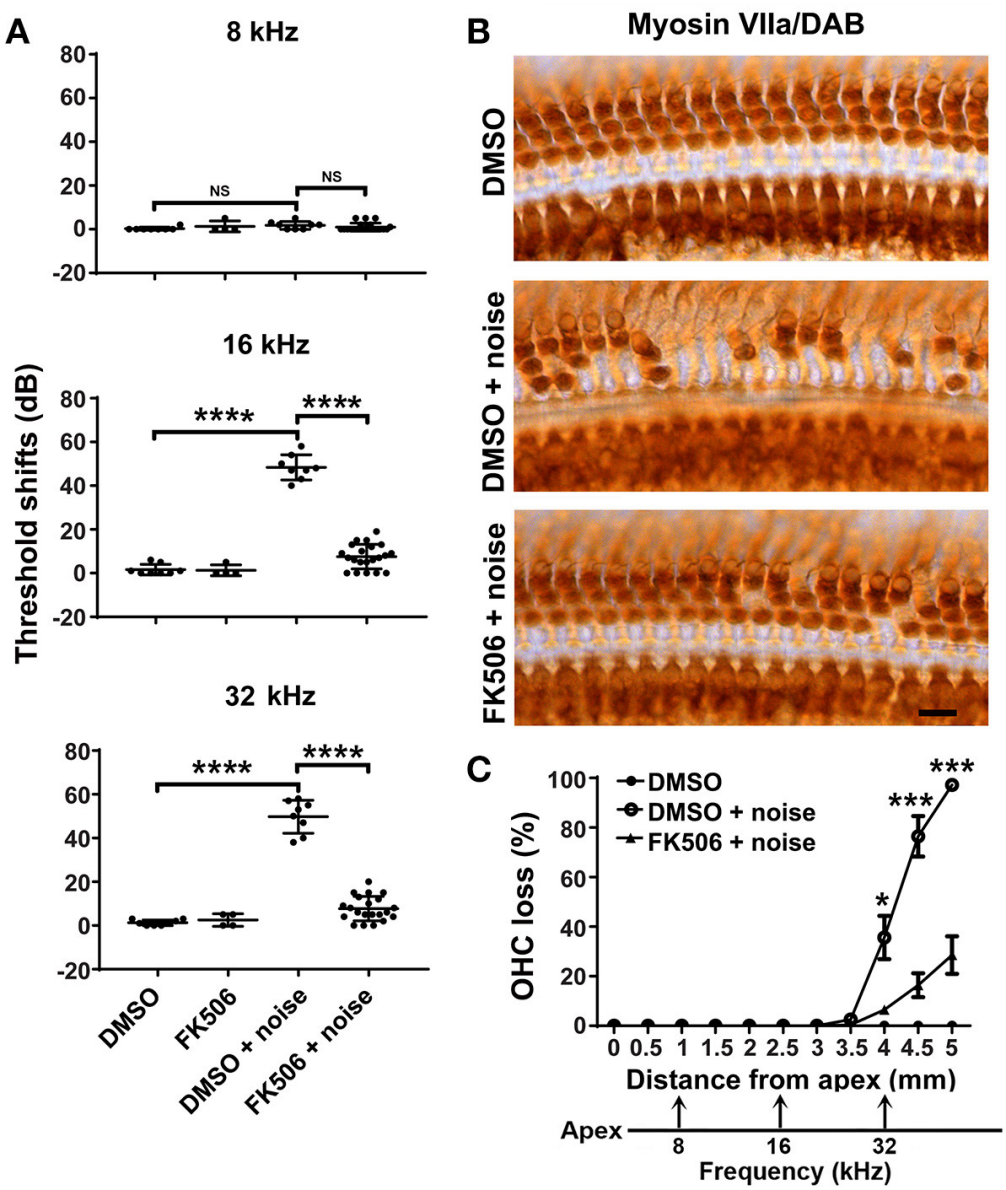
Treatment with FK506 prevents noise-induced outer hair cell (OHC) loss and hearing loss. **(A)** Treatment with FK506 significantly attenuates noise-induced auditory threshold shifts at 16 and 32 kHz measured 14 days after exposure. FK506 alone and vehicle control (dimethyl sulfoxide, DMSO) alone do not alter auditory threshold shifts. Data are presented as individual points measured in the left ears with means ± SD for each group; ****p < 0.0001; ns, not significant. **(B)** Representative images show Myosin-VIIa-labeled and 3,3′-diaminobenzidine-stained surface preparations from three groups: DMSO without noise, DMSO + noise, and FK506 + noise. Images were taken from the basal turn around 4.5 mm from the apex. Scale bar = 10 μm. **(C)** The percentage of cochlear OHC loss assessed 14 days after noise exposure, with OHC loss beginning around 3.5 mm from the apex and increasing until reaching 100% OHC loss in the basal region (5 mm from the apex). Treatment with FK506 significantly reduced noise-induced OHC loss. DMSO alone without noise exposure had no effect on hair cell loss. The distances along the cochlear spiral correlating with the frequencies 8, 16, and 32 kHz are indicated. Data are presented as means ± SEM for the left ears; DMSO only: *n* = 6, DMSO + noise: *n* = 9, FK506 + noise: *n* = 6; *p < 0.05, ***p < 0.0001.

Based on prior literature (Uemaetomari et al., 2005), we tested two doses (3 and 5 mg/kg) of FK506 against NIHL in our preliminary studies, and both doses of FK506 attenuated PTS. Since the 5-mg/kg dose offered stronger reduction of NIHL, we used 5 mg/kg for all the FK506 experiments. Auditory thresholds of four groups (DMSO alone, FK506 alone, DMSO + noise, and FK506 + noise) at 8, 16, and 32 kHz were measured 5 days before (baseline) and 2 weeks after moderate-PTS-noise exposure, with significant differences at 16 [*F*_(3, 34)_ = 162.4, *p* < 0.0001] and 32 kHz [*F*_(3, 34)_ = 149, *p* < 0.0001], but not at 8 kHz [*F*_(3, 37)_ = 1, *p* = 0.38], as analyzed by one-way ANOVA. Noise exposure significantly increased the auditory threshold shifts at 16 (*p* < 0.0001) and 32 kHz (*p* < 0.0001) compared to control mice without exposure (Figure 2A). Treatment with FK506 significantly attenuated noise-induced PTS at both 16 (*p* < 0.0001) and 32 kHz (*p* < 0.0001) (Figure 2A). Additionally, DMSO alone as the vehicle did not attenuate moderate-PTS-noise-induced auditory threshold shifts. The auditory thresholds of mice treated with FK506 alone did not differ from those of control mice treated with the vehicle control (DMSO) alone.

To confirm the protective effect of FK506 against moderate-noise-induced hearing loss, we counted the number of OHCs on surface preparations labeled with Myosin-VIIa and stained with DAB. Noise-induced OHC loss was significantly attenuated by treatment with FK506 (Figure 2B). Counts of the total number of missing OHCs along the entire length of the cochlear spiral showed that the loss of OHCs followed a base-to-apex gradient, with OHC loss beginning 3.5 mm from the apex and increasing to complete OHC loss in the base of the cochlear epithelium. Treatment with FK506 significantly reduced the noise-induced OHC loss as analyzed by repeated-measures ANOVA followed by *post-hoc* tests [*F*_(1, 12)_ = 35.207, *p* = 0.000; for detailed *post-hoc* values, see Table 1 for Figure 2C]. In the basal portion 5 mm from the apex, OHC loss was reduced from 100 to about 20% (Figure 2C).

**Table 1:** *Post-hoc* analysis of outer hair cell loss (Figure 2E).

**Groups**	**Distance from apex (mm)**	***p*-value**	**Symbol**
100 dB + DMSO vs. 100 dB + FK506	3.5	*p* = 0.369	ns
	4	*p* = 0.034	^*^
	4.5	*p* = 0.000	^***^
	5	*p* = 0.000	^***^

* *< 0.05*,

*** *< 0.001*.

Furthermore, to test if treatment with FK506 can attenuate hearing loss from even stronger noise insults, we evaluated FK506 against sPTS-noise conditions. In agreement with our previous data, sPTS-noise exposure induced auditory threshold shifts at all three tested frequencies (8, 16, and 32 kHz), with loss of both OHCs and IHCs 14 days after exposure. Treatment with FK506 significantly attenuated sPTS at only 8 kHz (*t*_12_ = 2.865, *p* = 0.0142), but not at 16 and 32 kHz (data not shown). These results suggest that treatment with FK506 can significantly attenuate noise-induced hearing loss in a noise-intensity-dependent manner. The potency of preventive effects was reduced with increasing noise-exposure intensity.

### Treatment With FK506 Diminishes Noise-Induced Accumulation of ROS

**Figure 3:**
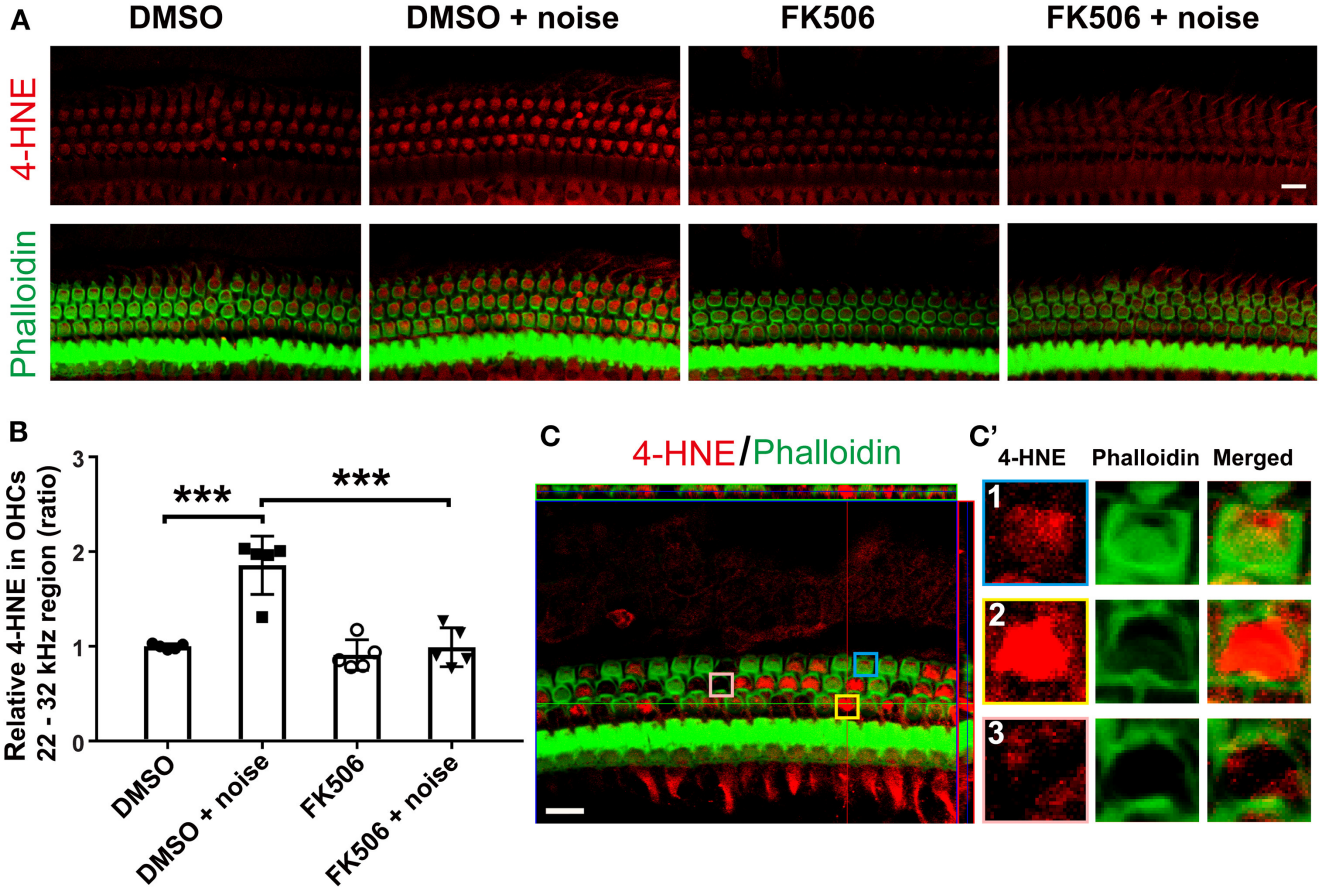
Treatment with FK506 blocks the induction of lipid peroxidation product 4-HNE by noise. **(A)** Surface preparations show immunolabeling for 4-HNE (red) in outer hair cells (OHCs) assessed 3 h after the completion of exposure. Noise-increased 4-HNE is inhibited by treatment with FK506. Green, phalloidin labeling of sensory hair cells. Images were taken from the basal turn corresponding to sensitivity to 22–32 kHz, and each figure is representative of five individual mice for each condition. Scale bar = 10 μm. **(B)** Quantification of relative 4-HNE immunolabeling intensity in grayscale in OHCs normalized to dimethyl sulfoxide control mice confirms inhibition by treatment with FK506. Data are presented as individual points with means ± SD; each condition was examined in one ear per mouse; ***p < 0.001. **(C)** This representative image shows very strong 4-HNE immunolabeling in structurally damaged OHCs assessed 3 h after exposure. The image was taken from the lower basal turn corresponding to sensitivity to 45 kHz. **(C′)** For better visualization, three OHCs were enlarged and presented in the right panels with intact OHCs (1), structurally damaged OHCs (2), and scars of lost OHCs (3). This figure is representative of five individual animals per group. Scale bar = 10 μm.

Accumulation of ROS in noise-induced OHC death is well documented in the literature, including in publications from our lab and others, through evaluation of markers of lipid peroxidation and protein nitration with 4-HNE and 3-NT, respectively (Yamashita et al., 2004; Fetoni et al., 2015; Wu et al., 2020a). To test whether treatment with FK506 prevents NIHL *via* inhibition of noise-induced accumulation of ROS, we assessed immunolabeling for 4-HNE, a lipid peroxidation product and a consequence of ROS formation acting as a surrogate marker for ROS, in OHCs 3 h after moderate-PTS-noise exposure. In agreement with previous reports, noise exposure increased immunolabeling for 4-HNE in basal turn OHCs, and this increase was significantly inhibited by treatment with FK506 (Figure 3A). Semi-quantitative analysis of immunolabeling for 4-HNE in grayscale in OHCs in the basal turn corresponding to sensitivity to 22–32 kHz confirmed a significant increase after exposure (*t*_8_ = 6.233, *p* = 0.0003), whereas treatment with FK506 significantly inhibited noise-increased 4-HNE (*t*_8_ = 5.243, *p* = 0.0008, Figure 3B). Treatment with FK506 alone showed similar levels as those of DMSO alone. Additionally, noise exposure did not increase immunolabeling for 4-HNE in OHCs in the apical and middle turns. It is worth mentioning that we did observe OHC loss 3 h after the PTS-noise exposure in the lower basal turn (about 4.5–5 mm from the apex). In this region, immunolabeling for 4-HNE was extremely strong in structurally damaged OHCs, but not in scars of lost OHCs (Figures 3C,C′, enlarged OHCs in the right panel).

### Treatment With FK506 Activates Autophagy in Cochlear Outer Hair Cells

**Figure 4:**
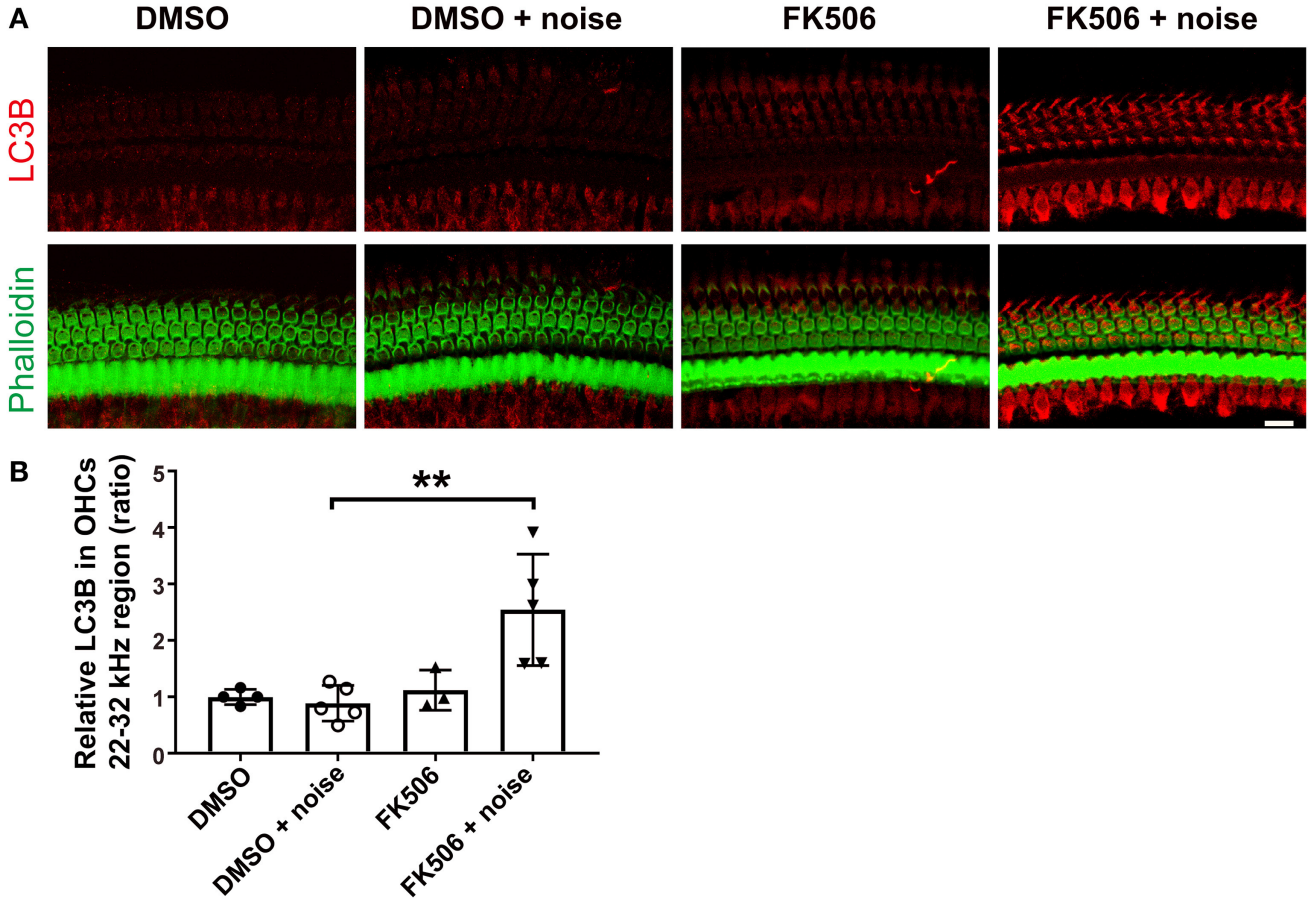
Treatment with FK506 increases LC3B in outer hair cells (OHCs). **(A)** Surface preparations show that immunolabeling for LC3B (red) in OHCs was increased after treatment with FK506 when assessed 3 h after the completion of noise exposure. Green, phalloidin labeling of sensory hair cells. Images were taken from the basal turn corresponding to sensitivity to 22–32 kHz, and each figure is representative of three to five individual mice for each condition. Scale bar = 10 μm. **(B)** Quantification of relative LC3B immunolabeling intensity in grayscale in OHCs normalized to dimethyl sulfoxide control mice confirms enhancement by treatment with FK506. Data are presented as individual points with means ± SD; each condition was examined in one ear per mouse; **p < 0.01.

Based on the literature, FK506 activates the autophagy system (Kim et al., 2017; Wang et al., 2017). To determine if autophagy plays a role in the prevention of noise-induced hearing loss by treatment with FK506, we assessed immunolabeling for LC3B in OHCs 3 h after moderate-PTS-noise exposure. In agreement with our previous publication (Yuan et al., 2015), noise exposure (DMSO + noise) did not change the levels of LC3B in OHCs compared to DMSO control mice without exposure when assessed 3 h after exposure, whereas treatment with FK506 increased immunolabeling for LC3B in OHCs in the basal turn compared to noise exposure alone (FK506 + moderate PTS noise vs. DMSO + moderate PTS noise; Figure 4A). Semi-quantitative analysis confirmed significant changes in the basal turn corresponding to sensitivity to 22–32 kHz (*t*_8_ = 3.570, *p* = 0.0073, Figure 4B). There were no differences in LC3B expression in OHCs between the three groups (DMSO alone, FK506 alone, and DMSO + moderate PTS noise). These data indicate that treatment with FK506 induces autophagy after noise exposure.

### Inhibition of Autophagy by Silencing LC3B Reduces the Protective Effect of FK506 Against Noise-Induced Hearing Loss and Outer Hair Cell Loss

**Figure 5:**
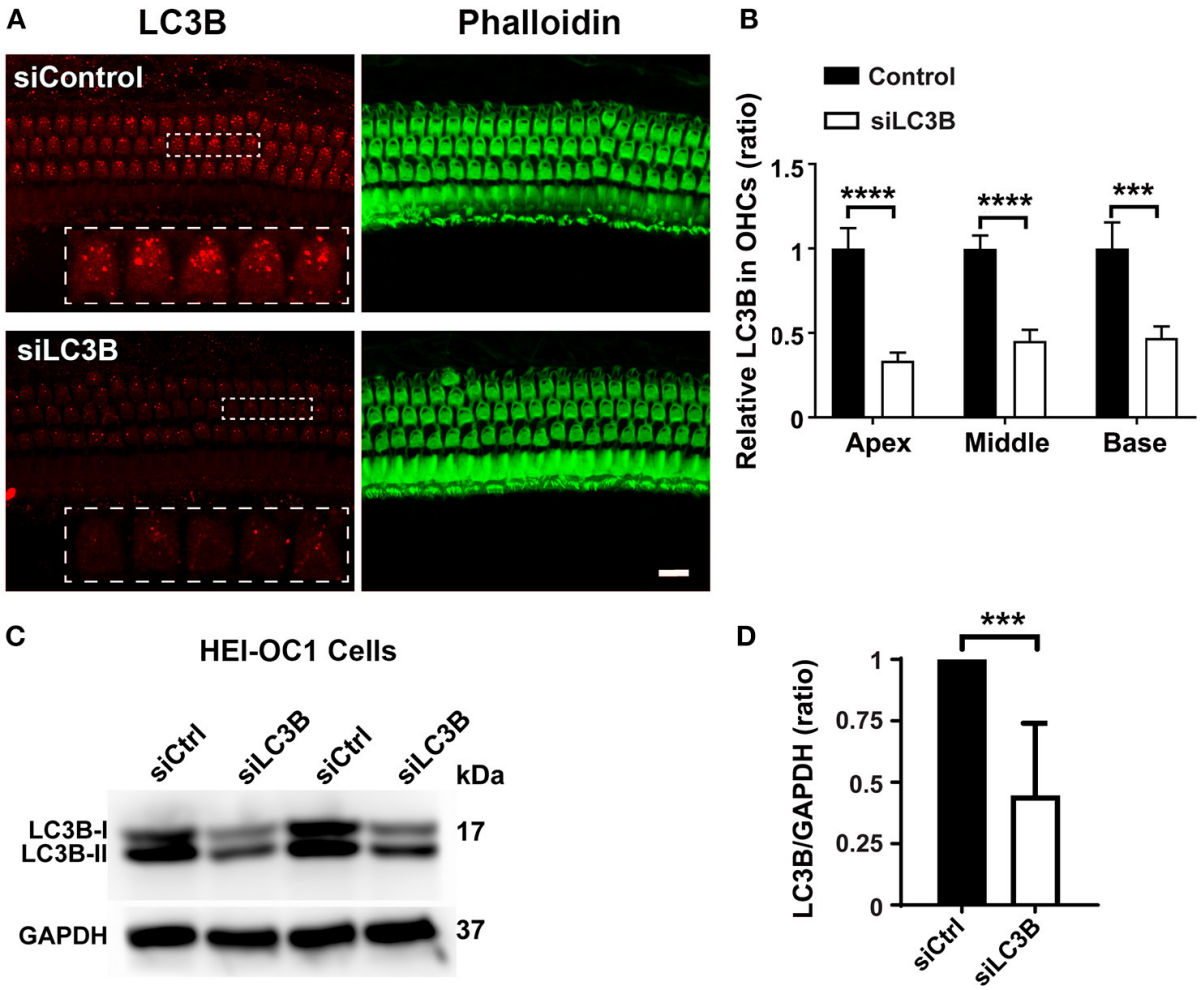
Downregulation of LC3B by LC3B-siRNA both *in vivo* and *in vitro*. **(A)** Surface preparations immunolabeled with LC3B reveal decreased immunolabeling for LC3B (red) in outer hair cells (OHCs) assessed 72 h after the intra-tympanic delivery of LC3B-siRNA. The representative images were taken from the basal turn corresponding to sensitivity to 22–32 kHz. The apical and middle turns showed a similar attenuation of LC3B. Green, phalloidin staining for sensory hair cells. Scale bar = 10 μm. **(B)** Quantification of LC3B immunolabeling intensity in grayscale in OHCs in the apical (corresponding to sensitivity to 8 kHz), middle (corresponding to sensitivity to 16 kHz), and basal turns (corresponding to 22–32 kHz) confirms a significant decrease. Data are presented as means ± SD, *n* = 4; ***p < 0.001, ****p < 0.0001. **(C)** Representative blots show that the protein levels of LC3B decreased after 48 h of transfection with the LC3B-siRNA in cells compared to the control group transfected with scrambled siRNA (siCtrl). GAPDH serves as a sample loading control. **(D)** Semi-quantification of the band density (both LC3B I and II) confirms a significant decrease. Data are presented as means ± SD; *n* = 4, ***p < 0.001.

**Figure 6:**
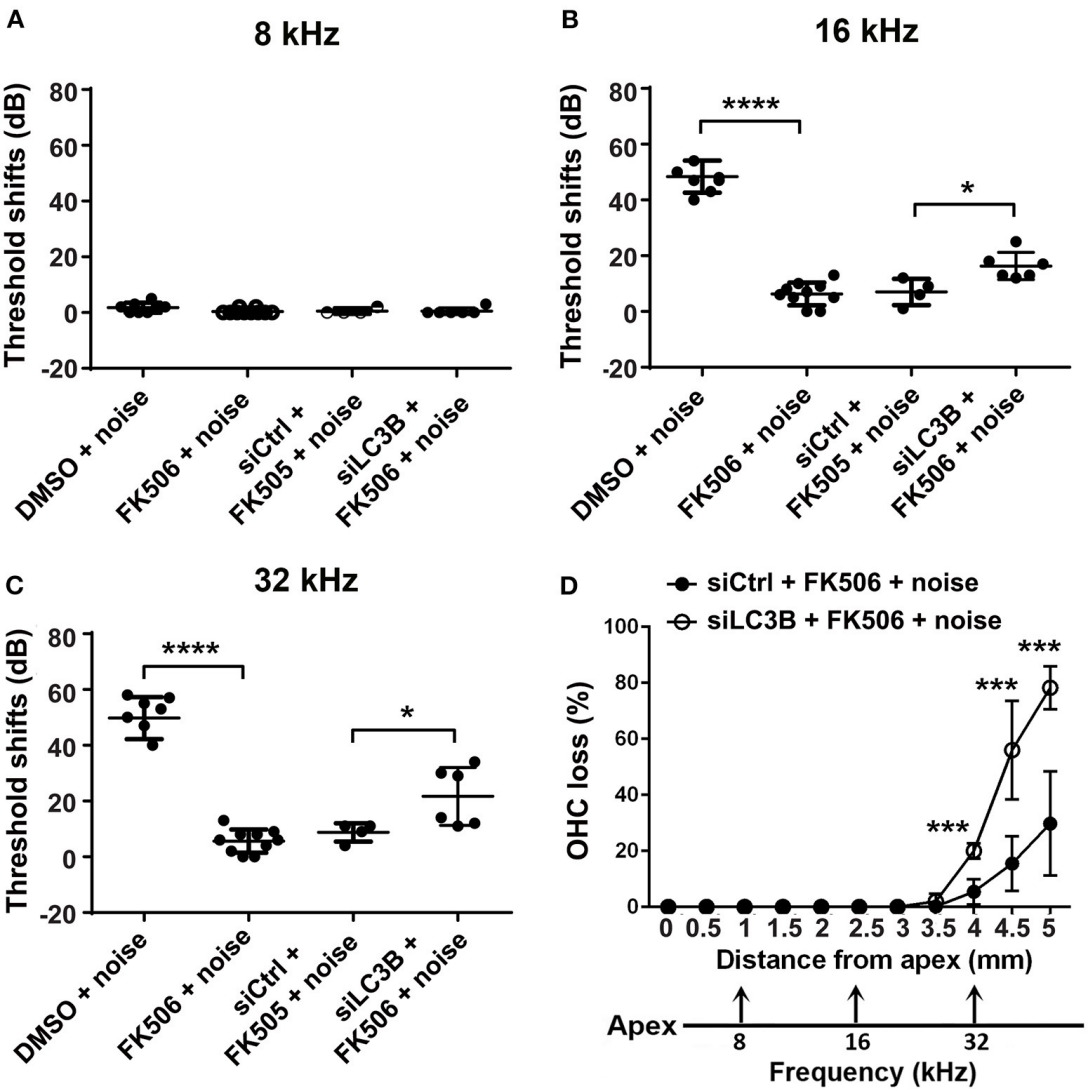
Inhibition of autophagy reduces the protective effects of FK506 against noise-induced hearing loss and auditory outer hair cell (OHC) loss. **(A–C)** Pretreatment with siLC3B reduces the protective effect of FK506 at 16 and 32 kHz when assessed 14 days after exposure. Noise does not induce auditory threshold shifts at 8 kHz. Data are presented as individual points for each mouse and means ± SD for each group; *p < 0.05, ****p < 0.0001. **(D)** Quantitative analysis of loss of OHCs along the entire cochlear spiral shows that the reduction of OHC loss by treatment with FK506 was partially diminished by pretreatment with siLC3B assessed 14 days after exposure. The distances along the cochlear spiral correlating with the frequencies 8, 16, and 32 kHz are indicated. Data are presented as means ± SD. siControl + FK506 + noise, *n* = 4; siLC3B + FK506 + noise, *n* = 6; **p < 0.01, ****p < 0.0001.

To confirm if autophagy is involved in the protective effects of FK506, we took advantage of LC3B siRNA techniques to reduce the expression of LC3B in OHCs and then evaluated the protective effect of FK506 against PTS-noise-induced auditory threshold shifts and hair cell loss. Based on our previous results, we selected the 0.6-μg dose of siLC3B or siControl delivered onto the RWN of the left ear of each mouse *via* intra-tympanic application (Oishi et al., 2013; Yuan et al., 2015). Immunolabeling for LC3B in OHCs on surface preparations was assessed nearly 72 h after siLC3B delivery (Figure 5A). Semi-quantification of the relative immunolabeling for LC3B converted to grayscale in OHCs of the apical, middle, and basal turns showed around 50% reduction compared to that of the siControls (apex: *t*_6_ = 10.13, *p* < 0.0001; middle: *t*_6_ = 10.66, *p* < 0.0001; base: *t*_6_ = 6.228, *p* = 0.0008, Figure 5B). Additionally, we transfected siLC3B to HEI-OC1 cells and analyzed silencing efficiency by Western blot (Figure 5C). Densitometry analysis of both LC3B-I and LC3B-II bands together showed about 50% reduction after silencing with siLC3B (*p* = 0.0095, Figure 5D). In agreement with our previous results (Yuan et al., 2015), pretreatment with siLC3B only mildly increased moderate-PTS-noise-induced auditory threshold shifts at 16 kHz by less than 10 dB measured 14 days after exposure, with no effect seen at 32 kHz compared to mice treated with siControl. There was no exacerbation of loss of OHCs with pretreatment of siLC3B after moderate PTS-noise exposure. Additionally, there were no differences in auditory threshold shifts between these three groups (moderate PTS noise, surgery + moderate PTS noise, and siControl + moderate PTS noise) when application of surgery or administration of siControl was performed 72 h before exposure to noise. Finally, after 72 h of siRNA pretreatment (left ears), both siControl and siLC3B mice received identical FK506 (5 mg/kg) treatment and noise exposure as described in Figure 2. The auditory threshold shifts (left ears) of mice pretreated with siLC3B were 10 dB greater at 16 (*t*_8_ = 3.004, *p* = 0.017) and 32 kHz (*t*_8_ = 2.362, *p* = 0.046) than those of the siControl mice (Figures 6A–C). To confirm that treatment with FK506 prevented NIHL in these mice, we assessed right-ear auditory threshold shifts in both groups. In agreement with our results described in Figure 2, the right-ear auditory threshold shifts of both groups were similar to the previous treatment with FK506 and significantly attenuated NIHL at both 16 (*p* < 0.0001) and 32 kHz (*p* < 0.0001).

Furthermore, counting OHCs along the entire cochlear spiral showed that pretreatment with siLC3B significantly decreased the protective effects of FK506 against OHC loss. In the siLC3B group, noise-induced OHC loss increased in the basal portions between 3.5 and 5 mm from the apex by repeated-measures ANOVA [*F*_(1, 11)_ = 45.040, *p* = 0.000; for detailed *post-hoc* values, see Table 2 for Figure 6D]. At 5 mm from the apex, the siLC3B group showed around 80% OHC loss compared to 30% loss with the siControl mice (Figure 6D). These results indicate that, when autophagy was inhibited by pretreatment with siLC3B, the protective effect of FK506 was partially blocked, indicating that the activation of autophagy is involved in the mechanism of FK506 protection.

**Table 2:** *Post-hoc* analysis of outer hair cell loss (Figure 6D).

**Groups**	**Distance from apex (mm)**	***p*-value**	**Symbol**
siControl + 101 dB + FK506 vs. siLC3B + 101 dB + FK506	3.5	*p* = 0.078	ns
	4	*p* = 0.000	^***^
	4.5	*p* = 0.000	^***^
	5	*p* = 0.0001	^***^

*** *< 0.001*.

## Discussion

Consistent with and building upon previous reports (Minami et al., 2004; Uemaetomari et al., 2005), our results show that treatment with the CaN antagonist FK506 attenuates noise-induced loss of OHCs and, consequently, NIHL in adult CBA/J mice. Additionally, attenuation of moderate-PTS-noise-induced hair cell loss and hearing loss by FK506 is significantly stronger than treatment with autophagy agonist rapamycin or antioxidant N-acetylcysteine (NAC) as evaluated in our previous report (Yuan et al., 2015). CaN is activated by a sustained elevation in intracellular calcium levels, which has been shown to be a consequence of traumatic noise exposure (Fridberger et al., 1998; Oliver et al., 2001). In our study, the expression of NFAT3 in OHC nuclei is significantly increased in a time-dependent fashion after exposure to moderate PTS noise, in agreement with the notion that the NFAT transcription factor family can be activated by CaN. OHC death, as a consequence of increased nuclear NFAT3, is compatible with an earlier report that application of an NFAT inhibitor on explants attenuates gentamicin-induced hair cell death (Bodmer et al., 2016) and is in line with the recent report showing that *Nfatc3* (*NFAT3*) deficiency in mice attenuates ototoxicity by suppressing TNF-mediated hair cell apoptosis (Zhang et al., 2019). NFAT forms a cooperative complex with AP-1 or other bZIP proteins through its binding site (Macián et al., 2001). The cooperation of NFAT with AP-1 is required for the transcription of several different genes, including IL-3, IFN-γ, and FasL, and plays an important role in immune responses and determining cell fate (Rao et al., 1997; Macián et al., 2001).

In fact, ROS and inflammation may interplay (Fetoni et al., 2019), as accumulation of both ROS and cytokines has been well documented in noise-induced OHC death (Yamane et al., 1995; Shi et al., 2003; Yamashita et al., 2005; Fujioka et al., 2006; Le Prell et al., 2007; Dhukhwa et al., 2019; Fetoni et al., 2019; Frye et al., 2019). Our results support this notion as we show that the lipid peroxidation product 4-HNE, acting as a surrogate marker for ROS, is highly expressed in damaged OHCs in the basal turn after noise exposure that induces high-frequency hearing loss, while treatment with FK506 inhibits noise-induced accumulation of 4-HNE. Additionally, our previous publication demonstrated that the levels of autophagy in OHCs increase after TTS-noise exposure where no OHC loss occurred, while the levels of autophagy marker LC3B remain close to that of the unexposed control mice after moderate-PTS-noise and even after sPTS-noise exposure (Yuan et al., 2015). Consistent with those findings, our current results show no changes in LC3B in OHCs after moderate-PTS-noise exposure. However, treatment with FK506 activates autophagy as indicated by an increased expression of LC3B in OHCs in the basal turn concurrent with attenuation of NIHL. Indeed lower levels of ROS have the ability to induce cellular defense pathways such as autophagy as seen in brain injury and cortical neuron apoptosis and optic nerve degeneration (Rodriguez-Muela et al., 2012; Wang et al., 2012). The current results support our previous conclusion that low levels of oxidative stress caused by exposure to TTS-noise activate autophagy, which inhibits cell apoptosis and prevents hair cell loss by inhibiting the accumulation of ROS. On the other hand, moderate PTS noise and sPTS noise induce excessive oxidative stress, which may trigger cell death pathways, leading to sensory hair cell death (He et al., 2017; Wu et al., 2020b). Furthermore, this notion is supported by the fact that inhibition of autophagy by siLC3B pre-treatment reduces the protective effect of FK506 against noise-induced loss of OHCs and hearing function. FK506 has previously been reported to be an activator of autophagy. FK506 can activate the translocation of TFEB from the cytoplasm into the nucleus by binding to ATP6V1A and then induce autophagy (Kim et al., 2017). Such a mechanism of FK506 action is in line with studies showing that treatment with FK506 increases the survival rate of myocardial cells *via* activation of the autophagy pathway (Wang et al., 2017) and is consistent with the general notion that activation of autophagy plays an important role in cellular survival, particularly in stress conditions such as starvation and oxidative stress (Mizushima et al., 2008; Esclatine et al., 2009; Rabinowitz and White, 2010). Our current results delineate that pretreatment with siLC3B mildly reduces the protective effects of FK506 against noise-induced OHC loss and hearing loss. This result is consistent with our previous data showing that pretreatment with siLC3B increased moderate-PTS-noise-induced auditory threshold shifts only minimally, by about 10 dB, compared with mice treated with siControl (Yuan et al., 2015), i.e., autophagy promotes sensory hair cell survival only slightly under certain conditions. This conclusion is supported by the fact that pharmacological activation of autophagy alone is insufficient to counteract ROS generation after moderate-PTS-noise exposure (Yuan et al., 2015). Nevertheless, autophagy may play dual roles in both cell survival early after insults and cell death at later stages (Baehrecke, 2005), although we never observe activation of autophagy in sensory hair cells after moderate-PTS-noise or sPTS-noise exposure.

We should emphasize that attenuation of moderate-PTS-noise-induced hearing loss and hair cell loss by FK506 is significantly stronger than treatment with autophagy agonist rapamycin or antioxidant NAC (Yuan et al., 2015). Attenuation of moderate-PTS-noise-induced auditory threshold shifts at 16 and 32 kHz was on average 40 dB after treatment with FK506 compared to 10–15 dB with rapamycin or NAC treatment. Additionally, reduction of moderate-PTS-noise-induced loss of OHCs was roughly 50% higher with treatment with FK506 than treatment with rapamycin or NAC (Yuan et al., 2015). It is worth noting that exposure of 12-week-old CBA/J mice to moderate PTS noise induces permanent hearing loss with loss of OHCs; the damage is less severe than that from sPTS-noise conditions, which result in loss of both OHCs and IHCs. Treatment with FK506 prevents the majority of damage induced by exposure to moderate PTS noise but only mildly attenuates sPTS-noise-induced damage. This result agrees with the general concept that the more severe the damage, the harder it is to achieve physiologically meaningful protection. This fact could be related to the activation of multiple cell death pathways after exposure to higher-intensity sPTS noise, including the possibility that inhibition of apoptotic OHC death promotes necrotic-like cell death (Zheng et al., 2014). A more effective protection would require further understanding of noise-induced hearing loss pathways and focus on potentially synergistic protective effects. We are aware that moderate-PTS-noise-induced auditory threshold shifts may be influenced by numerous confounding factors, for instance, surgical opening of the middle ear that could activate protective (e.g., heat shock proteins) or damaging (e.g., cochlear inflammation) mechanisms. Appropriate controls are essential before drawing conclusions. In our experiments, noise exposure was performed 72 h after surgery on the left ears. Our results showed there were no differences in auditory thresholds between moderate PTS noise exposure and surgery plus moderate PTS noise exposure. In summary, our results are in agreement with the notion that the noise-induced increase in nuclear NFAT3 in OHCs is a downstream consequence of CaN activation and a target of FK506. Treatment with FK506 inhibits noise-accumulated ROS and promotes autophagy, suggesting that FK506 attenuates noise-induced trauma that occurs not only *via* inhibition of CaN activity but also through inhibition of ROS and activation of autophagy.

## Data Availability Statement

The original contributions presented in the study are included in the article/supplementary material; further inquiries can be directed to the corresponding author.

## Ethics Statement

The animal study was reviewed and approved by the Institutional Animal Care and Use Committee at the Medical University of South Carolina.

## Author Contributions

S-HS designed research. Z-HH, SP, H-WZ, and Q-JF performed research. Z-HH, KH, and S-HS analyzed data and wrote the paper. All authors contributed to the article and approved the submitted version.

## Conflict of Interest

The authors declare that the research was conducted in the absence of any commercial or financial relationships that could be construed as a potential conflict of interest.

## Abbreviations

ABR: auditory brainstem response
CaN/PP2B: calcineurin
DMSO: dimethyl sulfoxide
FK506: tacrolimus
FKBP12: FK506-binding protein
GFP: green fluorescent protein
IHC: inner hair cell
IP: intraperitoneal
LC3B: microtubule-associated protein light chain 3B
LC3-GFP mice: green fluorescent protein (GFP)-LC3 transgenic mice
NFATc4/NFAT3: nuclear factor of activated T-cells isoform c4
NIHL: noise-induced hearing loss
OHCs: outer hair cells
PBS: phosphate-buffered saline
PBS-T: PBS with 0.1% Tween 20
PTS: permanent threshold shifts
sPTS: severe permanent threshold shifts
SDS-PAGE: sodium dodecyl sulfate polyacrylamide gel electrophoresis
siControl: scrambled small interfering RNA
siLC3B: LC3B small interfering RNA
SPL: sound pressure level
TDT: Tucker-Davis Technologies
TTS: temporary threshold shifts.
